# A Pilot Phase 1 Study of Intrathecal Pemetrexed for Refractory Leptomeningeal Metastases From Non-small-cell Lung Cancer

**DOI:** 10.3389/fonc.2019.00838

**Published:** 2019-08-30

**Authors:** Zhenyu Pan, Guozi Yang, Jiuwei Cui, Wei Li, Yu Li, Pengxiang Gao, Tongchao Jiang, Yanan Sun, Lihua Dong, Yuanyuan Song, Gang Zhao

**Affiliations:** ^1^Department of Radiation-Oncology, The First Hospital of Jilin University, Changchun, China; ^2^Department of Neuro-Oncological Surgery, The First Hospital of Jilin University, Changchun, China; ^3^Cancer Center, The First Hospital of Jilin University, Changchun, China; ^4^Department of Clinical Laboratory, The First Hospital of Jilin University, Changchun, China

**Keywords:** leptomeningeal metastases, non-small cell lung cancer, intrathecal chemotherapy, pemetrexed, refractory

## Abstract

**Objectives:** We aim to determine the feasibility, safety, maximally tolerated dose (MTD), recommended dose and potential anti-tumor activity of intrathecal pemetrexed (IP).

**Materials and Methods:** Lung adenocarcinoma patients with recurrent or progressive leptomeningeal metastases (LM) after intrathecal chemotherapy were recruited. IP dose was escalated from 10 mg. A minimum of three patients and a maximum of six were enrolled in each cohort. Schedule protocol was IP twice per week for 2 weeks in induction therapy, followed by once per week for 4 weeks in consolidation therapy. Serial samples of plasma and cerebrospinal fluid (CSF) were obtained for pharmacokinetic studies.

**Results:** Thirteen patients were enrolled between March 2017 and July 2018. *EGFR* driver oncogene was identified in most of the patients. Severe adverse events (AEs) were encountered in 31% (4/13) of the cases, including myelosuppression, radiculitis, and elevation of hepatic aminotransferases (EHA). Study protocol was revised due to lethal myelosuppression. Following protocol revision, vitamin B12 and folic acid supplementation was given at the beginning of treatment, and myelosuppression was well-controlled. Dose-limiting toxicities (DLT) were myelosuppression, radiculitis, and EHA. Two patients (2/2) developed dose-limiting myelosuppression at 15 mg level. One patient (1/6) experienced dose-limiting radiculitis and EHA at 10 mg level. MTD was 10 mg. Response rate was 31% (4/13) and disease control rate was 54% (7/13). The drug concentration showed a decreasing trend in serial CSF samples following each IP. After IP, the peak plasma concentration was reached at 4 h in two cases, 6 h in two cases, 9 h in one case, and 12 h in one case, respectively.

**Conclusion:** Pemetrexed was appropriate for intrathecal administration. IP at 10 mg dose in combination with vitamin supplementation on the schedule of 1–2 times per week showed controllable toxicity and good efficacy. This regimen paves the way for subsequent clinical trial.

**Clinical Trial Registration:** www.ClinicalTrials.gov, identifier NCT03101579.

## Introduction

Up until now, intrathecal chemotherapy (IC) is one of the treatment options for leptomeningeal metastases (LM) (1). The superiority of IC is that the agents can penetrate the blood–cerebrospinal fluid (CSF) barrier directly and maximize drug exposure in CSF. On this basis, a higher drug concentration can be achieved in the CSF with better cytotoxic effects by a low dose of intra-CSF administration than by systemic administration. Despite a few affirmations on several agents (2–6), methotrexate (MTX), cytarabine (ara-C), and thioTEPA are still the most frequently utilized agents for IC. As only a few treatment approaches for LM are available, it is significant to find an efficacious, and safe agent for IC.

Similar to MTX, pemetrexed is also a type of cell cycle specific and antimetabolic antitumor drug that inhibits metabolism of folic acid, but also a multitargeted antifolate agent. Pemetrexed combined with platinum is considered as one of the first-line treatment options for advanced non-small cell lung cancer (NSCLC), especially those with a non-squamous histology. Patients without central nervous system (CNS) metastases receiving maintenance pemetrexed developed fewer CNS metastases than those on the other regimens (7). This implies that pemetrexed has the potential capacity to overcome CNS involvement. However, it has been approved that the distribution of pemetrexed into brain is limited (8), and the CSF penetration of pemetrexed was <2% of plasma after intravenous pemetrexed (9). Clinical studies on intravenous pemetrexed in patients with CNS metastases showed limited clinical response rate (CRR) (10, 11). The treatment of LM may be more effective with direct intrathecal administration of much lower doses of pemetrexed than with intravenous administration.

A rat intrathecal pemetrexed (IP) model has been established. That study following a 1 mg/kg dose of IP in rats demonstrated that high pemetrexed concentrations were maintained in CSF for a long time (12). It is of note that the pemetrexed concentration in CSF at 24 h after intrathecal injection was 0.143 μM, which was close to the median IC_50_ value in NSCLC cell lines (12). The cytotoxicity of pemetrexed has been previously demonstrated to be dependent on both concentration and exposure time (13). In terms of CSF pharmacokinetic characteristics, pemetrexed is ideal for intrathecal injection. Furthermore, it was shown that no evidence of neuronal cell damage in histopathologic analysis of a deceased rat after IP injection (12). To date, there is no report about CNS toxicity induced by pemetrexed. IP presented potential advantages in feasibility and safety. No study concerning IP in humans has been reported. We designed this pilot phase 1 trial applying the approach of intra-CSF administration to make direct distribution of pemetrexed into CSF for the treatment of LM.

## Materials and Methods

We evaluated the feasibility, safety, maximally tolerated dose (MTD), recommended dose, and potential antitumor activity of IP. The primary objectives were to characterize drug-related toxicities, define the MTD, and to determine recommended dose. The secondary objective was to examine treatment efficacy.

### Patients

There is so little information about IP that the inclusion criteria for this study were strictly limited. The primary cancer of participants should be non-squamous NSCLC as pemetrexed is the first line systemic chemotherapy agent. No regimen has been widely regarded as the second line IC for recurrent or progressive LM; in light of this, patients who had previously received IC with confirmed recurrent or progressive LM were recruited. Recurrence and progression were ascertained according to the Response Assessment in Neuro-Oncology (RANO) proposal criteria and European Association of Neuro-Oncology-European Society for Medical Oncology (EANO-ESMO) guidelines for LM disease referring to the following elements: (1) progressively deteriorative neurological deficits typically associated with LM for more than 1 week; (2) worsening LM-related neuro-imaging findings. Meanwhile, other diseases or treatment side effects that may cause these conditions were excluded. A central review was performed to assess the progression of LM disease.

Eligibility criteria were as follows: (i) patients diagnosed with recurrent or progressive LM from lung adenocarcinoma; (ii) patients had received IC with or without other LM-related treatment previously; (iii) patients aged 18–75 years; (iv) with no severe hepatic and renal dysfunction, a glomerular filtration rate (GFR) of >80 mL/min, white blood cell count of ≥ 3.5 × 10^9^/L, and platelet count of ≥ 100 × 10^9^/L; (v) those with no other severe chronic diseases; and (vi) those with no severe dyscrasia.

Exclusion criteria were: (i) patients with serious CNS disorders including severe encephalopathy, moderate or severe coma, and Glasgow coma score of <9 points; (ii) patients presented obvious CNS injury by previous IC, such as chemical meningitis; (iii) patients were treated with systemic chemotherapy or new molecular targeted agents within 2 weeks; or (iv) other reasons that were unsuitable for this study, including patients with lethal or extensive systemic diseases with few treatment options, psychiatric illness and poor compliance.

### Study Design and Treatment Regimen

This study was a phase 1, open-label, dose-escalation pilot clinical trial. For the majority of intra-CSF drugs, elimination by metabolic inactivation is virtually negligible, and the predominant mechanism is CSF bulk flow excretion (14). Moreover, in terms of drug administration into the intrathecal space, which has little subsequent distribution outside of the sub-arachnoid space, dosage should be adjusted based on compartment volume, and drug concentrations rather than body surface area among different species (15). Taking these factors into consideration, the concentration for IP can be calculated based on dosage and CSF volume, but regardless of metabolic factors. As previously described (16), total CSF volume (ventricular and subarachnoid) at middle adulthood (40–55 years) was approximately 250 mL, and higher in aged population. According to our clinical data record, median age of LM patients from lung adenocarcinoma was 55–60 yrs. Thereafter, we speculated that total CSF volume would be 250 mL approximately in many cases enrolled in this study. Besides, the molecular weight of pemetrexed is 597.49 Da. In a previous study, the median 50% inhibitory concentration (IC50) of pemetrexed was 0.191 μM (approximately 114 μg) in various NSCLC cell lines (17). Moreover, the recommended dose of pemetrexed for intravenous injection was 500 mg/m^2^. After intravenous pemetrexed at a dose of 500 mg/m^2^, peak plasma concentration of pemetrexed was in a range of 100–200 μg/mL (18). In another clinical trial (19), in presence of GFR > 80 mL/min, the peak plasma concentration was 131 μg/mL after intravenous pemetrexed at a dose of 500 mg/m^2^. Furthermore, an optimal starting dose of pemetrexed (5–10 mg) was established for human studies in a previous study of rat IP model (12). In this study, regardless of drug metabolism, a peak pemetrexed concentration of 40 μg/mL would be achieved at a dose of 10 mg injection into 250 mL CSF in the event of ideally complete drug diffusion, which was less than half of the median IC_50_ (114 μg) or the peak plasma concentration (131 μg/mL) by intravenous pemetrexed at a dose of 500 mg/m^2^. In conclusion, we selected 10 mg as the starting dose to achieve potential efficacy.

**Figure 1:**
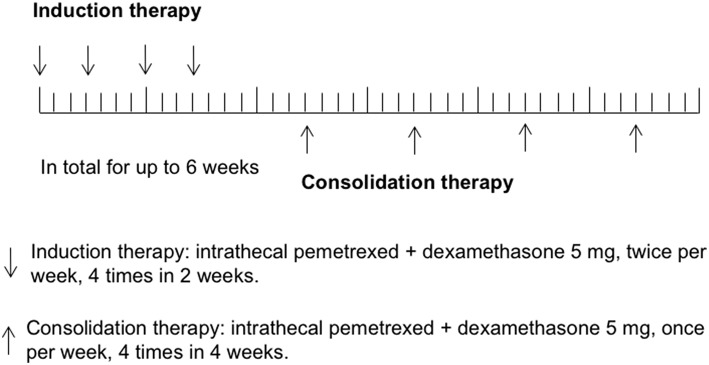
Study schema.

In the previous study of rat IP model establishing (12), pemetrexed was administered on a schedule of twice a week for 2 weeks via spinal canal injection through specifically designed indwelling subarachnoid catheters. In human CSF, half-lives of ara-C, thioTEPA, and MTX were <1 h, 3–4 h, and 4.5–8 h, respectively, (1). Similarly, half-lives of pemetrexed for the initial distribution/elimination and terminal elimination phase were 0.43 and 1.43 h in rat CSF (12). The most commonly recommended schedules of administration for intra-CSF agents (e.g., MTX, Ara-C, or thioTEPA) were twice weekly for 4 weeks, followed by once weekly for 4 weeks and then once per month. In this study, the regimen of IP consisted of induction therapy and consolidation therapy. Considering most participants received multiple cycles of IC previously, to avoid potential severe toxicity, induction therapy was given using twice IP per week for 4 times in 2 weeks, followed by consolidation therapy once per week for up to 4 times in 4 weeks. Total treatment time was not more than 6 weeks. Study schema was shown in Figure 1.

The initial dose of IP was 10 mg, escalated to 15 mg, and then 20 mg. A minimum of three patients and a maximum of six were enrolled in each cohort. A dose-limiting toxicity (DLT) was defined as grade 3 neurological toxicities (e.g., chemical meningitis) or other grade 4 toxicity. If none of the three patients experienced any DLT, the subsequent three patients were enrolled at the next higher dosage level. If one of three patients experienced a DLT, up to three more patients were enrolled at the same level. The MTD was defined as the dose where 0/3 or 1/6 patients experienced a DLT with at least two patients encountering DLT at the higher dose. If more than two patients experienced a DLT, that level was considered too toxic. The MTD was exceeded and an additional three patients should be treated at the next lower dose level.

Pemetrexed (Alimta, Eli Lilly, and Company) was administered by intrathecal injection via lumbar puncture. Pemetrexed lyophilized powder (100 mg) was dissolved in 0.9% sodium chloride solution (100 ml). The drug concentration of the solution was 1 mg pemetrexed per milliliters, and 10, 15, or 20 ml solution was taken for intrathecal injection. Dexamethasone (5 mg, 2 ml) was administered by intrathecal injection combined with pemetrexed simultaneously. Previous molecular target therapy was permitted to continue during this study of patients who had received the agents prior to the enrollment. In order to prevent the potential interference to this study, new antitumor agent was forbidden. Steroid and decreasing intracranial pressure agent were permitted to the patients with severe neurological deficits and increased intracranial pressure. Symptomatic therapy and supporting treatment were permitted to patients with severe conditions, including nutrition support, and maintaining electrolyte balance.

### Standard Protocol Approvals, Registrations, and Patient Consents

Procedures were compliant with the ethical principles of the Declaration of Helsinki and were approved by the Ethic Committee of the First Hospital of Jilin University. Prior to treatment, all patients or guardians signed an informed consent form before participating in the study. This study was registered in ClinicialTrials.gov (NCT03101579).

### Evaluation, Outcomes, and Follow-Up

Prior to treatment, following parameters were determined: general health conditions, Karnofsky performance score (KPS), neurological conditions, Glasgow coma score (GCS), CSF cytological examination, CSF biochemical test, a complete blood count and multichannel biochemical profiles. Imaging examination was used to evaluate systemic disease status. A standardized neurological examination, LM-related neurological symptoms and KPS record were performed prior to each IP. CSF cytology examination was performed using Thinprep plus Papanicolaou stain method every 2 weeks. Cerebrospinal MRI examination was performed before and after treatment using a scanner of 3.0 T field strength. LM-related imaging findings were recorded if abnormal leptomeningeal enhancement, subependymal enhancement, subarachnoid nodules, subependymal tumor spread, implantation metastases in vertebral canal, cranial nerve enhancement, hydrocephalus, and parenchymal metastases were identified on neuroimaging examination. AEs were evaluated by physical examination, neurological examination, CSF examination, complete blood count and multichannel biochemical profile monitoring 1–2 times per week according to Common Terminology Criteria for Adverse Events (CTCAE, version 4.03). More than grade 3 events were defined as severe AEs. The severe AEs would be recorded and reported to National Medical Products Administration and department of pharmacy of the hospital.

The clinical response was determined after the termination of treatment by three blinded neuro-oncologists according to RANO proposal criteria which were based on three basic elements (20). The determination of neurological assessment should consider the effect of steroid consumption. Improved or stable neurological assessment following increased steroid dose was eliminated. Neuroimaging assessment was performed according to the revised Leptomeningeal Assessment in Neuro-Oncology (LANO) grid (21) by three neuro-radiologists and two neuro-oncologists with the blind method. CSF cytology assessment was performed by three blinded cytopathologists. Follow-up physical, standardized neurological examinations and CSF cytological examinations were carried out every 2–3 months or at any instance of suspected clinical progression until death. Neurological progression-free survival (NPFS) was defined as time from the start of treatment until LM disease progression or death. LM disease progression was defined according to Response Assessment in Neuro-Oncology proposal criteria. Survival time was measured from the inclusion of this study until death or the last follow-up.

### Plasma and CSF Sample Collection

Plasma and CSF samples were collected and analyzed for drug concentration in the cases of signed informed consent. CSF samples were collected prior to each IP via lumbar puncture. Plasma samples were collected serially at different time points within 24 h after IP.

### Chemicals and Reagents

Pemetrexed and oxcarbazepine, with a purity of >99.0%, were purchased from the National Institute for the Control of Pharmaceutical and Biological Products (Beijing, China). Acetonitrile of high-performance liquid chromatography grade was purchased from Fisher Scientific (Fair Lawn, NJ, USA). Ultra-high purity water, prepared using the Milli-Q system, was used throughout the study. All other chemicals were of high-performance liquid chromatography grade.

### Measurement of Plasma and CSF Pemetrexed

Pemetrexed was detected using a liquid chromatography-tandem mass spectrometry system consisting of a Shimadzu LC-20ADXR high performance liquid chromatography system (Shimadzu, Kyoto, Japan) coupled to a Qtrap 5,500 mass spectrometer (Sciex, Ontario, Canada) equipped with a TurboIonSpray ion source. Parameters employed in the system were listed in Supplementary Table 1. Chromatography was performed on a Poroshell 120 SB-C18 column (50 × 4.6 mm, 2.7 μm) maintained at 40°C, and the flow rate was 0.9 mL/min. The mass spectrometry parameters were: nebulizer and heater gas flow rates: 50 L/min, curtain gas flow rate: 25 L/min, dwell time: 50 ms, ion spray voltage: 5,500 V, heater gas temperature: 500°C. Analyst Software (Shimadzu, Kyoto, Japan) was used for system control and data acquisition.

Frozen human plasma and CSF samples could thaw in a water bath at room temperature. Aliquots (100 μL) of plasma or CSF (or calibration standard or quality control sample) were added to 50 μL internal standard working solution (20 ng/mL in 50% methanol, oxcarbazepine). Then acetonitrile (300 μL) was added to precipitate protein. The mixture was vortex-mixed for 1 min and centrifuged for 5 min at 15,000 rpm. An aliquot (30 μL) of the supernatant after protein precipitation was injected into the liquid chromatography-mass spectrometry.

### Statistical Analysis

All outcome measures were assessed on intention-to-treat analysis. This is a phase I clinical trial.

## Results

### Patient Characteristics

Between March 2017 and July 2018, 13 patients (male: 4; female: 9; age: 37–71 years; median: 55 years) were enrolled in this study. All the patients were hospitalized. All participants presented progressively deteriorating neurological symptoms/signs typically associated with LM for more than 1 week. Five patients (patient 1, 2, 8, 12, and 13) showed worse neuroimaging. Median KPS was 30 (20–70). All participants presented positive CSF cytology. Twelve patients presented LM-related neuroimage findings and one with negative neuroimaging.

Eleven patients had received CNS involved-field radiotherapy combined with concomitant IC as the first line LM-related therapy in prior treatment. Eleven patients had received systemic therapy previously, including five with systemic chemotherapy and eleven with molecular target therapy. Ten patients with *EGFR* mutation had received tyrosine-kinase inhibitor (TKI) drugs. One patient with anaplastic lymphoma kinase (ALK) fusion gene had received ALK inhibitor drugs. Before this study, eight patients had undergone LM recurrence and had received salvage IC. Seven cases had received both of intrathecal MTX and ara-C. Five cases showed no clinical improvement to intrathecal MTX combined with ara-C. Three cases showed no clinical improvement to intra-MTX or intra-ara-C. Patient characteristics were shown in Table 1.

**Table 1:** Patients' general information.

**Patient no**.	**Sex**	**Age (years)**	**Tumor history (months)**	**Interval of initial LM diagnosis to the enrolment (months)**	**KPS**	**GCS**	**Neuroimaging findings**	**Prior LM-related treatment**	***EGFR*/*ALK* gene**	**Prior systematic treatment**	**Systematic treatment during this study**
P1	F	57	23.5	13.2	20	14	Linear enhancement	Concurrent IM and WBRT; IA; IMA	*EGFR* positive	GPx1, ACx5, Icotinib, Osimertinib	–
P2	F	37	13.2	8.2	20	15	Linear enhancement	Concurrent IM and WBRT; IA; IMA	*EGFR* positive	Icotinib, Osimertinib	–
P3	F	36	22	8.4	20	14	Nodules and linear enhancement	Concurrent IM and WBRT; IA; IMA	*EGFR* positive	Erlotinib, Osimertinib, APx2	Osimertinib (80 mg/day)
P4	M	66	3.2	3.2	70	15	Nodules and Linear enhancement	Concurrent IM and WBRT; IA	Not detected	–	–
P5	F	49	43	9.8	60	15	Nodules and Linear enhancement	Concurrent IM and WBRT; IMA	*EGFR* positive	Gefitinib, Icotinib, Osimertinib, APx4	Osimertinib (80 mg/day)
P6	F	55	7.2	7.2	20	13	Nodules and linear enhancement	Concurrent IM and WBRT	*EGFR* positive	Gefitinib, Osimertinib	Osimertinib (80 mg/day)
P7	F	38	19.4	14.4	40	15	Linear enhancement	Concurrent IM and WBRT; IA; IMA	*EGFR* positive	Icotinib, Osimertinib	–
P8	F	47	38.4	38.4	60	15	Linear enhancement	Concurrent IM and WBRT; IM	*EGFR* positive	Gefitinib, Icotinib, Osimertinib	Osimertinib (80 mg/day)
P9	M	71	26.5	0.5	20	10	Negative	IA	*EGFR* positive	Gefitinib, DPx7, APx3	–
P10	M	56	3.3	2.5	20	14	Nodules	Concurrent IM and WBRT	Not detected	–	–
P11	F	50	54.5	21.1	40	15	Nodules and Linear enhancement	Concurrent IM and WBRT; IMA	*EGFR* positive	Gefitinib, Icotinib, Osimertinib, APx4	Osimertinib (80 mg/day)
P12	M	39	18	9.6	70	15	Nodules and Linear enhancement	Concurrent IA and WBRT	*ALK* positive	Crizotinib, lorlatinib	Lorlatinib (100 mg/day)
P13	F	61	47	42	50	15	Linear enhancement	IM	*EGFR* positive	Erlotinib, Gefitinib, Osimertinib	Gefitinib (250 mg/day) and Osimertinib (80 mg/day)

*F, female; M, male; LM, leptomeningeal metastases; KPS, Karnofsky performance status score; EGFR, epidermal growth factor receptor; ALK, anaplastic lymphoma kinase; GCS, Glasgow Coma Scale; IM, intrathecal methotrexate; IA, intrathecal cytosine arabinoside; WBRT, whole brain radiotherapy; IMA, intrathecal methotrexate and cytosine arabinoside; IP, intrathecal pemetrexed; GP, gemcitabine and cisplatin; AC, pemetrexed and carboplatin; AP, pemetrexed and cisplatin; DP, docetaxel and cisplatin*.

### Treatment

**Figure 2:**
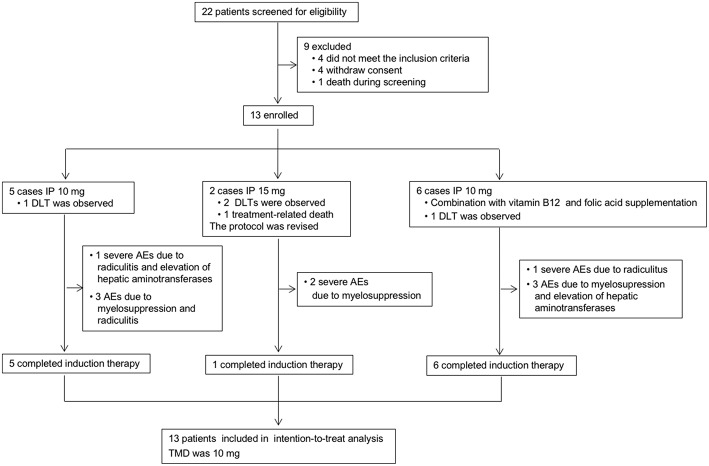
Study profile. IP, intrathecal pemetrexed; DLT, dose-limiting toxicity; AEs, adverse events; MTD, maximally tolerated dose.

The study profile was provided in Figure 2. Treatment information was shown in Table 2. A total of 72 times IP was given to all patients, with a median of 6 (2–8) times. Eleven cases (85%) completed the induction therapy. Two participants didn't complete induction therapy due to ineffectiveness or severe AEs. Patient 1 withdrew from the treatment after seven times IP due to systemic disease progression. Patient 2 underwent temporary cessation (7 days) due to grade 4 elevation of hepatic aminotransferases (EHA) and withdrew from the treatment due to presentation of grade 4 radiculitis after the fifth IP. Patient 3 withdrew from the treatment due to no neurological improvement after three times IP. Patient 4 completed induction therapy and quitted from treatment for personal reasons. Patient 6 withdrew from treatment due to hydrocephalus after six times IP. Patient 7 showed severe hematologic toxicity after two times IP, then withdrew from treatment and died 1 week later. Patient 11 had sudden onset of brain stem hemorrhage 3 days after the fourth IP and died 5 days later. Patient 12 presented involvement of parenchymal brain and withdrew from treatment after six times IP. Patient 13 withdrew from treatment after four times IP due to grade 3 myelosuppression, moderate fatigue and weight loss. The remaining four patients (patient 5, 8, 9, and 10) received eight times IP. Molecular target therapy was continued during this study in 9 patients who had received the molecular target agents prior to the enrollment.

**Table 2:** Treatment and outcomes.

**Patient no**.	**Dose level (mg)**	**Times of IP**	**KPS score**		**CSF cytology assessment**		**Neurological assessment**		**Neuroimaging assessment**	**Response evaluation**	**Death**	**Cause of death**	**NPFS (months)**	**Survival time (months)**
			**Before treatment**	**After treatment**	**Before treatment**	**After treatment**	**Neurological exam**	**Neurological symptoms**						
P1	10	7	20	90	positive	positive	Improved	Improved	Improved	Response	Yes	SDP	2.9	2.9
P2	10	5	20	70	positive	positive	Improved	Improved	Stable	Response	Yes	LMP	5.3	6.1
P3	10	3	20	20	positive	Not review	Stable	Stable	Not review	N/E	Yes	LMP	1.2	1.2
P4	10	4	70	90	positive	positive	Stable	Improved	Stable	Stable	Yes	LMP	5.9	7.2
P5	10	8	60	90	positive	positive	Stable	Improved	Stable	Stable	Yes	LMP	12.5	14
P6	15	6	20	20	positive	Not review	Stable	Stable	Worse	PD	Yes	LMP	1.0	2.1
P7	15	2	40	40	positive	Not review	Stable	Improved	Not review	N/E	Yes	AEs	0.3	0.3
P8	10	8	60	90	positive	positive	Stable	Improved	Improved	Response	Yes	LMP	10.2	12.5
P9	10	7	20	30	positive	positive	Stable	Improved	Not review	N/E	Yes	LMP	1.6	1.6
P10	10	8	20	70	positive	positive	Improved	Improved	Stable	Response	Yes	LMP	2.5	3.8
P11	10	4	40	50	positive	Not review	Stable	Improved	Not review	N/E	Yes	Stroke	0.6	0.6
P12	10	6	70	70	positive	positive	Worse	Worse	Stable	PD	Yes	LMP	1.0	6.9
P13	10	4	50	50	positive	positive	Stable	Improved	Stable	Stable	No	–	5.2	5.2

*IP, intrathecal pemetrexed; KPS, karnofsky performance status score; GCS, Glasgow Coma Scale; PD, progressive disease; SDP, systemic disease progression; LMP, leptomeningeal metastasis progression; AEs, adverse events; NPFS, neurological progression-free survival; N/E, non-evaluable*.

### Toxicities and AEs

The major AEs were myelosuppression, radiculitis and EHA (Table 3). Only one case presented grade 1 fatigue and grade 2 weight loss. No patient showed CNS toxicity and obvious mucositis. Total severe adverse events (AEs) were encountered in 31% (4/13) of the cases, including 2 with grade 4–5 hematological toxicities, and 2 with grade 4 radiculitis, and 1 with grade 4 EHA. At 10 mg dose level without initial vitamin supplementation, the incidence of AEs was 80% (4/5), including 1 with grade 3 hematological toxicities, 1 with grade 3 hematological toxicities, grade 4 radiculitis and grade 4 EHA, 1 with grade 1–2 radiculitis, and 1 with grade 1 hematological toxicities. The incidence of severe AEs was 20% (1/5), including 1 with radiculitis and EHA. At 15 mg dose level, all the 2 patients suffered severe AEs of grade 4–5 hematological toxicities. At 10 mg dose level with initial vitamin supplementation, the incidence of AEs was 67% (4/6), including 1 with grade 2 hematological toxicities and grade 4 radiculitis, 1 with grade 1–2 radiculitis, 1 with grade 2 EHA, 1 with grade 3 hematological toxicities and grade 2 radiculitis. The incidence of severe AEs was 17% (1/6), including 1 with grade 4 radiculitis.

**Table 3:** Toxicities and AEs.

**Patient no**.	**Dose level (mg)**	**AEs**	**Grade of CTCAE**	**Time point of AE occurrence**	**Management**
P1	10	Myelosuppression (Leucopenia and thrombocytopenia)	III	After the 1st IP	rhG-CSF; rhTPO
P2	10	Myelosuppression (Leucopenia); Elevation of hepatic aminotransferases. Radiculitis (transient paraplegia).	III IV IV	After the 1st IP After the 4th IP After the 5th IP	rhG-CSF; Glutathione, monoammonium glycyrrhizinate, bicyclol Spontaneous recovery
P3	10	–	–	–	–
P4	10	Radiculitis	I-II	After the 5th IP	None
P5	10	Myelosuppression (Leucopenia and thrombocytopenia)	I	After the 3rd IP	None
P6	15	Myelosuppression (Leucopenia and thrombocytopenia)	IV	After the 3rd IP	rhG-CSF; rhTPO
P7	15	Myelosuppression (Leucopenia and thrombocytopenia)	V	After the 2nd IP	rhG-CSF; rhTPO
P8	10	Myelosuppression (Leucopenia and thrombocytopenia) Radiculitis (transient paraplegia)	II IV	After the 2nd IP After the 8th IP	rhG-CSF; rhIL-11 Spontaneous recovery
P9	10	–	–	–	–
P10	10	Radiculitis	I-II	After the 4th IP	None
P11	10	–	–	–	–
P12	10	Elevation of hepatic aminotransferases	II	After the 3rd IP	Glutathione, monoammonium glycyrrhizinate, bicyclol
P13	10	Radiculitis Myelosuppression (Leucopenia and thrombocytopenia) Fatigue Weight loss	II III I II	After the 3rd IP After the 3rd IP After the 3rd IP After the 3rd IP	None rhG-CSF None None

*Adverse events (AEs) were evaluated according to the Common Terminology Criteria for AE (CTCAE, version 4.03). No, number; IP, intrathecal pemetrexed; rhG-CSF, recombinant human granulocyte colony-stimulating factor; rhIL-11, recombinant human interleukin-11; rhTPO, recombinant human thrombopoietin*.

Myelosuppression commonly occurred after 1–3 times IP. Six cases showed thrombocytopenia. Four of them with grade 3 or more were treated by recombinant human interleukin-11 and/or recombinant human thrombopoietin, and 3 cases showed remission about 1–2 weeks later. Seven patients presented leukopenia. Five of them with grade 3 or more received recombinant human granulocyte colony stimulating factor, and 4 cases showed recovery about 3–5 days later. One patient presented severe myelosuppression (leucopenia and thrombocytopenia) after two times of IP at 15 mg level. Finally, the patient died 1 week later. Thereafter, protocol was revised accordingly. Before protocol revision, four (57%) showed hematologic toxicities of grade 3 or more. However, only one (17%) patient presented grade 3 hematologic toxicities with initial vitamin supplementation after protocol revision. It indicated that myelosuppression could be significantly ameliorated by vitamin supplementation at 10 mg dose level.

EHA was noticed in two cases after two times of IP. Hepatic aminotransferases continually increased after the following IP. One patient exhibited grade 4 after the fourth IP. No parenchymal lesion was found in the liver by abdominal CT and ultrasonography examination. Agents including glutathione, monoammonium glycyrrhizinate, and bicyclol were given. Concentration of transaminase showed gradual decrease in 2–3 weeks, and recovery within 1 to 2 months.

Two cases presented grade 4 radiculitis about 1 h later after the fifth and eighth IP, respectively, which was manifested as the symptoms of nerve root irritation, including loss of tactile, pain, and warm sensation in the part beneath the bilateral hips, a myodynamia of level 0, and absence of pathological signs. The symptoms were spontaneous remission in 5–6 h. No related toxicity was observed in follow-up.

### MTD

At the beginning of this study, two cases showed grade 3 myelosuppression after the first IP at a dose of 10 mg. Myelosuppression showed remission after symptomatic treatment. Afterwards, folic acid and vitamin B12 were given to the patients, and hematologic toxicity did not reappear. Meanwhile, one of them showed grade 4 radiculitis and grade 4 EHA defined as the DLT. Then three more cases were enrolled and showed no toxicity of grade 3 or more at 10 mg level. The dose level was escalated to 15 mg. A participant showed grade 4 myelosuppression 3 days after the third IP and was recovery after symptomatic treatment. No hematologic toxicity reappeared after folic acid and vitamin B12 supplement. Another participant presented severe myelosuppression after the second IP. Then the patient quit from treatment and died 1 week later. The initial two cases presented DLT at 15 mg level. The study was suspended because of a lethal event.

The myelosuppression was controllable in most cases by symptomatic treatment and did not relapse after folic acid and vitamin B12 supplement. Besides, most of the participants showed improved neurological symptoms/signs. After taking these into consideration, the study continued, and the protocol was revised by the approval of Ethics Committee as follows. Vitamin B12 and folic acid supplementation was given at the beginning of IP. Folic acid (400 μg, quaque die) was given daily until 21 days after the last IP. Single dose of vitamin B12 (1,000 μg, via intramuscular injection) was given at the first IP. The dose level decreased to 10 mg, and then six cases were enrolled at 10 mg level. Only one patient presented grade 3 myelosuppression. Another patient showed grade 4 nerve root toxicity which was defined as DLT. Eventually, one patient (1/5) showed DLT of radiculitis and EHA at the 10 mg level without initial vitamin supplementation. Two patients showed DLT of hematological toxicities at 15 mg level. Then one patient (1/6) showed DLT of radiculitis at the 10 mg level with vitamin supplementation. Therefore, the MTD was 10 mg.

### Clinical Response Evaluation

Response evaluation and outcomes were shown in Table 2. For each participant, neurological examination and LM-related neurological symptoms and KPS record was performed by a single examiner to minimize exam variability. Improved disease based on neurological assessment is defined by a change of 2 or more levels in a given domain (e.g., gait) or alternatively by a change to level 0 in any one domain of the RANO proposal neurological examination instrument. Neurological assessment was improved in 3 patients (patient 1, 2, and 10), stable in 8 and worse in 1 (patient 12). Improved neurological dysfunction included gait and consciousness. Except two (patient 3 and 7), 11 patients showed relief from LM-related symptoms for at least 2 weeks. CSF cytology remained positive in the other 10 patients. Three patients (patient 3, 6, and 7) did not review CSF cytological examination due to severe AEs, hydrocephalus, and stroke. Eight participants reviewed MRI after treatment. The other 5 participants reviewed CT scan due to severe conditions and MRI intolerance. In neuroimaging assessment, 2 patients (patient 1 and 8) with leptomeningeal linear enhancement were evaluated as improved. Six patients were stable. Patient 6 was evaluated as worse neuroimaging due to hydrocephalus by CT scan according to the LANO grid. The remaining 4 patients were not evaluable due to absence of MRI review.

The clinical response is determined by three blinded neuro-oncologists according to RANO proposal criteria which were based on three basic elements. Four patients were assessed as response, including 2 cases (patient 1 and 8) with improved neuroimaging assessment, improved or stable neurological examination and stable CSF cytology, the remaining two cases (patient 2 and 10) with improved neurological examination, stable neuroimaging assessment and stable CSF cytology. Three patients (patient 4, 5, and 13) assessed as stable disease based on stable neurological exam, stable neuroimaging assessment and stable CSF cytology. Two patients (patient 6 and 12) assessed as progressive disease based on worse neurological examination and worse neuroimaging assessment, respectively. Remaining 4 participants without MRI review after treatment were not evaluable.

On intention-to-treat analysis, total CRR was 31% (4/13). Total disease control rate (DCR) was 54% (7/13). The CRR was 33% (2/6) and 29% (2/7) for patients with or without initial vitamin supplementation, respectively. DCR were 50% (3/6) and 57% (4/7) for patients with or without initial vitamin supplementation, respectively. At 10 ml dose level, CRR was 36% (4/11) and DCR was 64% (7/11).

### Follow-Up and Survival

After the treatment of this study, seven patients continued the administration of previous molecular target therapy that had been applied prior to this study. Patient 1 received anti-angiogenic target therapy for systemic disease progression of lymphangitis carcinomatosa in both lungs but showed no response. Patient 12 received bevacizumab for brain edema.

All patients were followed at least 4 months until death or January 5, 2019. The median neurological progression-free survival was 2.5 months (0.3–12.5 months). The median survival was 3.8 months (0.3–14 months). Twelve patients (92%) died, among which 10 (83%) died from cancer progression including 9 (75%) with LM and CNS involvement progression and one (8%) with systemic disease progression. One case (8%) died from treatment-related toxicities. One case (8%) died from non-cancerous disease (Table 2).

### Drug Concentration in Plasma and CSF Samples

**Figure 3:**
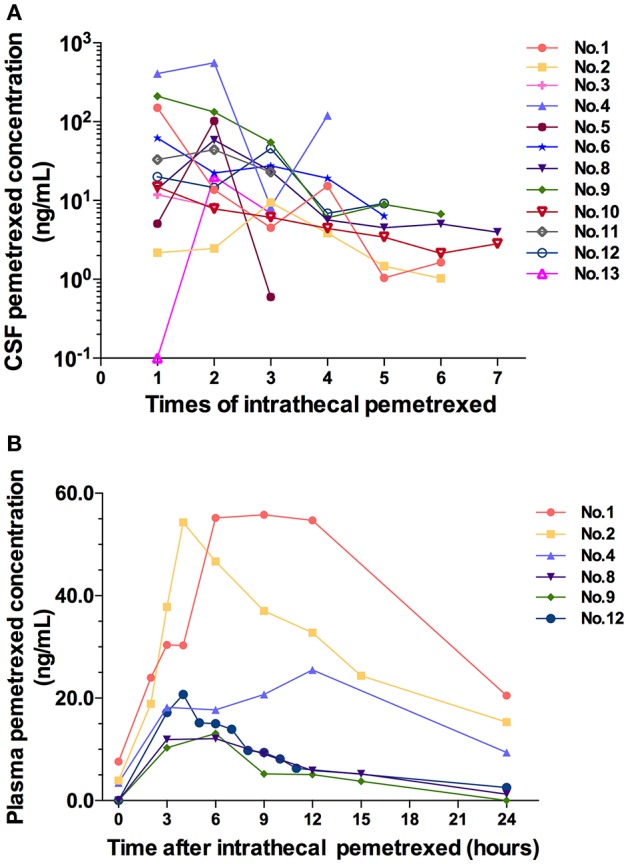
**(A)** CSF pemetrexed concentration after each time of intrathecal pemetrexed. **(B)** Plasma pemetrexed concentration at different time points after intrathecal pemetrexed. No, patient number.

All cases accepted CSF samples collection and analysis of drug concentration. The drug concentration showed a decreasing trend in serial CSF samples following each IP (Figure 3A). After the fourth IP, 8 (8/9) patients showed a CSF drug concentration of ≤ 19.2 ng/ml. After the fifth IP, the CSF drug concentration in the 8 patients (8/8) was of ≤9.16 ng/ml. After the sixth IP, 6 patients (6/6) showed CSF drug concentration of ≤6.75 ng/ml.

Six cases in 10 mg dose level accepted blood samples collection and analysis. Their blood samples were collected within 24 h after the third or fifth IP. After IP, the peak plasma concentration was reached at 4 h in two cases, 6 h in two cases, 9 h in one case, and 12 h in one case, respectively (Figure 3B). The plasma concentration values from 3 patients (patient 2, 8, and 12) can be used to analyze the half-life for terminal elimination phase. The terminal half-lives of pemetrexed in plasma were 11.51, 5.24, and 8.42 h in 3 cases, respectively.

## Discussion

To the best of our knowledge, this is the first clinical trial of IP. Pemetrexed presented feasibility of intrathecal administration. Hematologic toxicities were the main IP-related AEs which were controllable at 10 mg level combined with vitamin supplementation. IP at 10 mg dose level on the schedule of regimen administration in this study showed satisfactory CRR. The pilot pharmacokinetic studies showed that 10 mg dose on a schedule of 1–2 times per week was an appropriate intrathecal administration regimen for pemetrexed without accumulation in CSF. This study recommends pemetrexed at 10 mg dose level on the schedule of 1–2 times per week as an intrathecal administration agent for LM disease.

Several previous studies demonstrated that high dose intravenous injection pemetrexed showed therapeutic benefits in treating relapsed primary central nervous system lymphoma (CNSL) and secondary central nervous system lymphoma (22–24). Lymphoma is a chemotherapy-sensitive tumor. Furthermore, it suggests that high dose intravenous injection pemetrexed may have better drug penetration in the cases with CNS lymphoma caused by local blood-brain barrier disruption. However, in a prospective study on high-dose pemetrexed for brain or leptomeningeal metastases (10), patients were treated with intravenous pemetrexed at doses of 500, 750, 900, and 1,050 mg/m^2^. Pemetrexed distributed from the plasma to the CSF with the resulting CSF concentrations <5 % of plasma. Limited anti-tumor activity was seen, which might be related to low CSF concentrations (10). It also indicated that the blood-CSF barrier was not disrupted by LM. In this study, low dose pemetrexed injected directly into CSF presented well-antitumor activity.

CSF drug concentration showed a tendency of decline following the treatment. CSF drug concentration of the several cases in initial 4 times IP fluctuated at high values. Meanwhile, the drug concentration presented large different among different patients. However, after the fourth IP, CSF drug concentration fluctuated in a small range. Meanwhile, the difference of drug concentration between different patients decreased. We speculated that the various degrees of pathophysiological conditions in different patients affecting the distribution and elimination of pemetrexed led to the difference of drug concentration in different cases as well as high values and the fluctuation of drug concentration. Following the effective treatment, the distribution or elimination of pemetrexed was normalized gradually by improved disease conditions. Therefore, both of the values fluctuation and the difference of CSF drug concentration among different patients decreased. It was suggested that not only was the IP treatment efficacious, but also the schedule of 1–2 times per week was an appropriate intrathecal administration regimen for pemetrexed without accumulation in CSF.

Plasma half-life of pemetrexed for the terminal elimination phase was 2.45 h in the previous study of rat IP model (12). In this study, plasma half-life of the 3 patients was relatively prolonged, and half-life values from 3 patients showed significant difference. We speculated that the difference of plasma half-life in 3 cases was also caused by various degrees of pathophysiological conditions. The distribution/elimination of pemetrexed was interfered with by the pathophysiological state. The abnormal distribution/elimination of pemetrexed in CSF resulted in an extended time from CSF into the blood, thus extending the half-life of the plasma terminal, and causing the difference in 3 cases.

Myelosuppression is the most common DLT of systemic administration of pemetrexed, manifesting predominantly as neutropenia, with nadir around day 10, and recovery generally by day 15 (25). Thrombocytopenia and anemia are relatively uncommon with an incidence of <10% (26, 27). As a type of regional chemotherapy, dosage of IP was far less than with systemic chemotherapy. We did not anticipate that such a low dose IP could induce such severe hematologic toxicities. Folic acid and vitamin B12 supplementation had been approved to reduce the incidence of intravenous pemetrexed induced AEs without affecting treatment efficacy in previous studies (25, 28). To avoid potential interferences, folic acid and vitamin B12 supplementation was not designed into the beginning of this study. Duration of vitamin supplementation prior to pemetrexed had no correlation with incidence of pemetrexed-related toxicities (29). This suggests that pemetrexed-based chemotherapy does not have to be delayed accommodating a schedule of vitamin supplementation. In this study, vitamin supplementation was proved to reduce the incidence of IP induced myelosuppression. Nevertheless, we cannot explain the reason of severe hematologic toxicities, especially the high incidence of thrombocytopenia.

EHA is the most common non-hematologic toxicity of systemic pemetrexed chemotherapy (26). Grade 1/2 hepatic enzyme elevations occurred in 60–70% of patients and were commonly transient, with recovery to baseline by the beginning of the subsequent cycle (26). Approximately 12% of patients presented grade 3 abnormalities. Grade 4 liver function abnormalities were extremely rare (26). In this study, EHA was one of the DLT. We speculated that EHA is partially attributed to the frequency of pemetrexed regimens. The regimen of systemic administration was one cycle every 3 weeks. However, the regimen of induction IP was twice per week. The interval of IP regimen was extremely shorter than that of systemic administration. It may hamper hepatic metabolism, which resulted in persistent EHA. Despite the low incidence rate, it is still necessary to monitor liver function periodic during IP treatment. Additionally, considering it was usually transient in systemic administration, no management was given to the patients with EHA at the beginning. However, aminotransferase elevation was persistent after following IP. Symptomatic treatment was necessary.

There are some limitations in this study. First, intraventricular administration was not applied in this study due to risks inherent in reservoir implantation surgery and catheter-related complications as well as high medical and nursing expenses. It was difficult to obtain serial CSF samples without an indwelling subcutaneous access device. The half-life and clearance rate of pemetrexed in cerebrospinal fluid could not be analyzed. Matched samples of CSF and blood were not obtained for pharmacokinetic analysis. Second, due to serious condition, some patients did not receive MRI examination on time, and the neuroimaging information was not comprehensive.

Despite the limitations, this study revealed that pemetrexed was a novel intrathecal drug which showed controllable AEs and promising efficacy for LM patients, even those with few treatment options. In a recent phase 1 clinical study on LM arising from NSCLC (30), a high proportion achieved a response with AZD3759 in both CNS lesions (83%) and extracranial disease (72%) in patients never received TKI agents, but the confirmed response was only 14% in patients received TKI treatment previously. It indicated that the potential use of this drug as monotherapy in the first-line setting for *EGFR*-mutant NSCLC. In this study, patients previously received IC with confirmed relapse or progressive LM were recruited. Furthermore, most of the patients had received more than one TKI or ALK agents. In these refractory patients, satisfactory response and disease control were achieved. That indicates pemetrexed as a multi-targeted antifolate agent exhibits good antitumor effect in CSF. IP at a recommended dose of 10 mg on a schedule of 1–2 times per week paves the way for subsequent clinical trial.

## Data Availability

The datasets generated for this study are available on request to the corresponding author.

## Author Contributions

ZP: conception and design. LD and GZ: administrative support. ZP, GY, LD, JC, WL, PG, and TJ: provision of study materials or patients. ZP, GY, PG, TJ, and YSu: collection and assembly of data. ZP, GY, YSo, and YL: data analysis and interpretation. ZP, GY, and YSo: manuscript writing. GY and ZP: clinical trial registration. All authors: final approval of manuscript and accountable for all aspects of the work.

### Conflict of Interest Statement

The authors declare that the research was conducted in the absence of any commercial or financial relationships that could be construed as a potential conflict of interest.
