# Supplementary material for: A Pilot Phase 1 Study of Intrathecal Pemetrexed for Refractory Leptomeningeal Metastases From Non-small-cell Lung Cancer
Source: Front Oncol. 2019 Aug 30;9:838. doi: 10.3389/fonc.2019.00838 (PMC6730526; doi:10.3389/fonc.2019.00838)
Supplement: Supplementary file 1 [file Table_1.DOC]

Supplementary Table 1

Parameters of LC-MS/MS

| Compound | Ionization Mode | m/z | DP (eV) | CE (eV) | Solvents for gradient elution | | Gradient conditions |
| --- | --- | --- | --- | --- | --- | --- | --- |
|  |  |  |  |  | A | B | (B concentration %) |
| Pemetrexed | ESI (+) | 428.3/281.2 | 80 | 24 | 0.1% formic acid | acetonitrile | 0 min: 20%  →1.0 min: 20%  →1.5 min: 70%  →2.0 min: 70%  →3.1 min: 20%  →5.0 min: 20% |
| Oxcarbazepine (IS) |  | 253.1/208.0 | 70 | 26 |  |  |  |

IS, internal standard; DP, declustering potential; ESI, electrospray ionization; CE, collision energy.
